# Effects of exercise based on ACSM recommendations on anxiety in children and adolescents: a meta-analysis of randomized controlled trials

**DOI:** 10.3389/fphys.2025.1744254

**Published:** 2026-01-14

**Authors:** Jinhua Xian, Qing Hu, Bo Jiang

**Affiliations:** School of Educational Science, Jiangsu Second Normal University, Nanjing, China

**Keywords:** adolescents, anxiety, children, exercise, meta-analysis

## Abstract

**Purpose:**

To investigate the effects of different dosages of exercise on anxiety symptoms in children and adolescents.

**Methods:**

The present study screened randomized controlled trials (RCTs) from PubMed, Embase, Web of Science and Cochrane Library databases. According to the suggestions of American College of Sports Medicine (ACSM), all included studies were categorised into a high and a low/uncertain adherence group. The random-effects model was adopted in the meta-analysis. Subgroup analyses were also conducted to explore the differences in outcomes.

**Results:**

A total of 27 RCTs including 2022 participants were extracted and included for analysis. The results indicated that exercise interventions may have an anxiolytic effect in youth (SMD = −0.36, 95% CI: −0.58 to −0.15, p = 0.0009). According to the ACSM, 13 studies were classified into high adherence group, and 14 studies were classified into low/uncertain adherence group. Subgroup analysis showed that the anxiety reduction was significantly larger in high ACSM adherence group (SMD = −0.67, 95% CI: −1.10 to −0.23, p = 0.002) than in low/uncertain ACSM adherence group (SMD = −0.13, 95% CI: −0.33 to 0.07, p = 0.21). Furthermore, exercise interventions longer than 11 weeks showed significantly greater effects than those shorter than 11 weeks. Only interventions delivered at least three times per week and incorporating combined exercise modalities exerted anxiolytic effects. Moreover, exercise interventions significantly reduced anxiety symptoms in populations with physical illnesses.

**Conclusion:**

The meta-analysis demonstrated that exercise interventions showed significant anxiolytic effects in children and adolescents. Moreover, the anxiety reduction in the high ACSM adherence group was significantly larger than that in the low/uncertain ACSM adherence group.

## 1 Introduction

Anxiety is an emotion featuring the dimensions of apprehension and somatic symptoms of tension (American Psychological Association, 2025). In terms of prevalence, anxiety is a frequently encountered mental health problem which tends to correlate with lower academic performance (Mazzone et al., 2007), diminished quality of life and social functioning among younger populations (Haller et al., 2014; Leichsenring and Leweke, 2017). In our review, the age of children and adolescents was set at 6–18 years old, according to the World Health Organization (2020). Therefore, anxiety should be identified early and promptly treated in this age group (Asselmann and Beesdo-Baum, 2015; Hill et al., 2016).

Currently, mainstream interventions for anxiety include psychological therapy and pharmacotherapy (Craske and Stein, 2016). As cognitive behavioral therapy, a commonly used form of psychological therapy, has several limitations such as high cost of treatment (Marciniak et al., 2005) and long waiting time before treatment (Chartier-Otis et al., 2010). Selective serotonin reuptake inhibitors are the mainstay in pharmacotherapy, which are often linked to a variety of adverse effects including metabolic effects, dependence and withdrawal reactions (Baldwin et al., 2005; Taylor et al., 2012; Bandelow et al., 2015). In addition, the social stigma of both psychological and pharmacological treatments are often responsible for deterring individuals from seeking help (Curcio and Corboy, 2020).

Due to the limitations of mainstream treatments for anxiety, exercise has gradually attracted attention as an intervention with no social stigma and low cost (Pascoe et al., 2020). A meta-analysis showed that interventions involving exercise significantly reduce anxiety in college students, and aerobic exercise has the greatest effect (Lin and Gao, 2023). Other meta-analyses also explored whether mind–body exercise could decrease anxiety levels in different populations (So et al., 2020; Lin et al., 2024). When exploring exercise’s anxiolytic effects in patients with clinical anxiety, it was found that aerobic exercise markedly reduced anxiety levels, and the programs of higher intensity outperformed those of lower intensity (Aylett et al., 2018). As for intervention involving exercise in adolescents, there were two meta-analyses. The study found that exercise moderately reduces anxiety in the population (Carter et al., 2021), while the other one found that resistance training reduces anxiety in adolescents (Marinelli et al., 2024). The previous meta-analyses have extended the age limit to 25–26 years (Carter et al., 2021; Marinelli et al., 2024), which may consequently obscure developmental heterogeneity and make inferences under children and adolescents. Further, in the absence of evidence-based exercise dose framework, it is not clear how the exercise dose is related to alleviation of anxiety in this population.

To address these gaps, we conducted a meta-analysis of randomised controlled trials involving participants that are under the age of 18 years and the exercise dose is categorized according to the American College of Sports Medicine (ACSM) guidelines (Garber et al., 2011). Furthermore, based on the suggestions of the ACSM, we divided the included studies into a high and a low/uncertain adherence group and compared their respective effects on anxiety in children and adolescents. This study explores how different exercise dosages impact anxiety symptoms, thereby supplying critical evidence to inform the development of exercise intervention approaches in diminishing anxiety among children and adolescents.

## 2 Methods

This meta-analysis followed the Preferred Reporting Items for Systematic Reviews and Meta-Analyses (PRISMA) guidelines and has been registered in PROSPERO (CRD420251175738).

### 2.1 Search strategy

PubMed, Embase, Web of Science and Cochrane databases were searched from inception to 30 August 2025 without language limits. The strategy was constructed according to the PICOS format and included four concept blocks connected with the Boolean operator AND: (1) population (children OR adolescents), (2) intervention (exercise OR physical activity OR sport), (3) outcome (anxiety OR anxiety disorder), and (4) study design (randomized OR randomized controlled trial OR RCT). MeSH and free-text terms were employed; the synonyms that comprise each block will be joined with the operator OR. The full search strategy is given in Supplementary Appendix S1.

### 2.2 Eligibility criteria

Inclusion criteria: (1) individuals under the age of 18; (2) an experimental group engaging in any form of exercise; (3) a control condition that included waitlist, no treatment, control group with no exercise intervention, and treatment (physical activity) as usual; (4) anxiety symptoms assessed with validated and standardised psychological scales; (5) randomized controlled trial (RCT) design, including cluster RCTs; and (6) no language restriction.

Exclusion criteria: (1) individuals aged over 18 years; (2) non-randomized controlled trials (non-RCTs); (3) studies lacking available data on outcome indicators; and (4) unpublished or grey literature.

Two researchers independently screened studies by reviewing titles and abstracts. Subsequently, full texts were assessed to establish their eligibility to be included in the meta-analysis. Disagreement was solved by discussion.

### 2.3 Data synthesis and analysis

Two researchers independently extracted information from included studies based on specific inclusion criteria. First author, country, health status, age, sample, type of intervention, control condition, and outcome measures were extracted. After the extraction of the data, the extent of exercise dose and adherence were rated individually by each researcher and the intervention elements were coded based on the ACSM domains (cardiorespiratory, resistance, and flexibility exercise) (Garber et al., 2011). Interventions expressly recommending two or more ACSM domains were categorized as combined exercise and further sub-categorized into cardiorespiratory-flexibility, resistance-flexibility, cardiorespiratory-resistance, and all three components; disagreements were resolved through discussion. In line with previous meta-analyses (Fang et al., 2024; Zhao et al., 2024), we scored the compliance of each RCT on a 0–2 scale for frequency, intensity/workload and duration of cardiorespiratory, resistance and flexibility exercises (2 = completely compliant, 1 = uncertain, 0 = non-compliant). The proportion of exercise doses meeting ACSM recommendations was then calculated for each study. Studies achieving adherence rates of ≥75% to the ACSM-recommended doses were categorized as high adherence, while those with rates <75% were classified as low/uncertain adherence (Table 1).

**TABLE 1:** ACSM recommended dosage adherence assessment criteria for exercise intervention.

Exercise dose	Cardiorespiratory exercise	Resistance exercise	Flexibility exercise
Frequency	3–5 days per week	2–3 days per week	≥2–3 days per week, daily
Intensity/workload	40%–60% VO_2_R or HRR; RPE of 12–13 on a 6–20 scale	Start with 40%–50%1RM, more capable with 60%–70% 1RM	Stretch until you feel your muscles being pulled tight or a slight discomfort
Duration	Continuous or cumulative 30 min	≥1 group, 8–12 repetitions	Keep static pulling for 10–30 s; repeat 2–4 times

VO_2_R: oxygen uptake reserve, HRR: heart rate reserve, RPE: rating of perceived exertion.

### 2.4 Statistical analysis

Multi-arm trials were narrowed to one arm of the single exercise meeting the ACSM dose to prevent a possibility of duplicating the shared control group, and yield one contrast of intervention *versus* control. The scores of anxiety measured post-intervention were also extracted and then analysed using Review Manager 5.4. The effect size was expressed as SMD, with its 95% confidence interval (CI). According to the ACSM compliance criteria, studies included in this analysis were divided into a high adherence group (compliance rate ≥75%) and a low/uncertain adherence group (compliance rate <75%). Given heterogeneity in the characteristics of exercise interventions, which include length, frequency, and duration, a random-effects model was used in the meta-analysis. To assess potential publication bias, funnel plots were constructed, and Begg’s rank correlation test and Egger’s linear regression test were performed to evaluate the symmetry of the funnel plots.

### 2.5 Quality appraisal

Following the Cochrane Handbook (Higgins et al., 2011), two researchers conducted independent assessments of the risk of bias (ROB) across random sequence generation, allocation concealment, blinding of participants and personnel, blinding of outcome assessment, incomplete outcome data, selective reporting, and other bias, each of which was categorized into low, unclear, and high risk.

## 3 Results

### 3.1 Selection results

**FIGURE 1:**
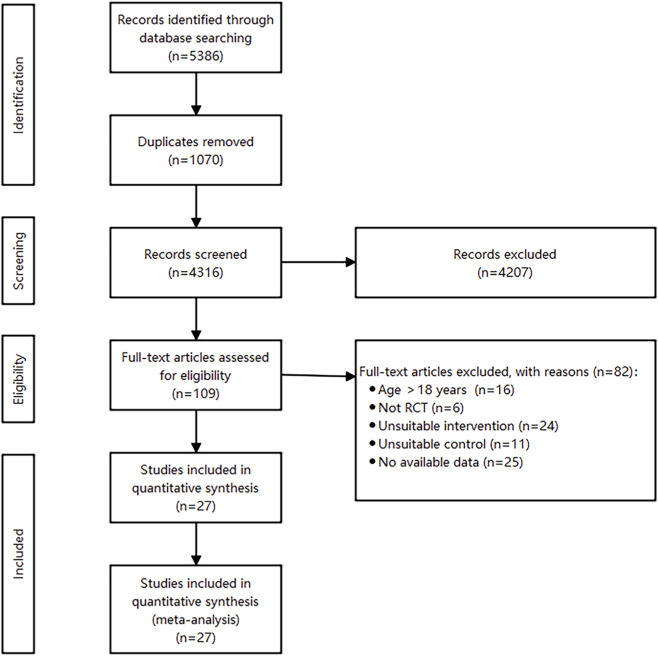
Flow chart of literature selection.

The study selection process was shown in Figure 1, a total of 5,386 articles were retrieved from PubMed, Embase, Web of Science and Cochrane Library databases. After removing duplicate records, 4,316 studies remained. 109 articles were selected for further evaluation following the screening of titles and abstracts. After reviewing the full texts, 27 studies were included in the meta-analysis.

### 3.2 Study characteristics

This study included 27 randomized controlled trials with 2022 participants: 1,083 in experimental groups and 939 in control groups (Table 2). Regarding participant characteristics, 12 studies involved healthy populations, 8 studies focused on participants diagnosed with physical illnesses (i.e., obesity, type 1 diabetes, and irritable bowel syndrome), and 7 studies targeted individuals with mental illnesses (i.e., anorexia nervosa, ADHD, depression, and autism spectrum disorder). All included studies employed exercise interventions: 6 cardiorespiratory, 10 flexibility, and 11 combined exercise (cardiorespiratory-resistance n = 2; cardiorespiratory-flexibility n = 2; resistance-flexibility n = 1; all three components n = 6). Ranging from 3 to 24 weeks, the interventions were conducted 1 to 7 times weekly, with each session lasting 10–90 min. As for the control group conditions: 9 studies involved a no intervention control, 5 studies used a waitlist control, 7 studies employed non-exercise interventions, and 6 studies implemented treatment as usual. In addition, only four of the 27 RCTs assessed cortisol (Nazari et al., 2020; Rodrigues et al., 2021; Gehricke et al., 2022; Heidarianpour et al., 2023), with a significant post-intervention reduction reported in one study (Heidarianpour et al., 2023).

**TABLE 2:** Characteristics of included studies.

Author (year)	Country	Health status	Age Mean (SD)	Total/male/female	Intervention	Control	Outcome
Akko et al. (2020)	Germany	Healthy	T + C: 9.35 (0.6)	T:27/NR C:21/NR	Cardiovascular exercise Length of intervention: 10 weeks Freq: 3 times a week Duration: 45 min	CON	STAI
Bazzano et al. (2022)	USA	Healthy	NR	T: 42/NR C: 44/NR	Yoga Length of intervention: 8 weeks Freq: one time a week Duration: 45 min	WL	SCARED
Carei et al. (2010)	USA	Mental illness (eating disorders)	T + C: 16.52 (2.35)	T:26/NR C:27/NR	Yoga Length of intervention: 8 weeks Freq: 2 times a week Duration: 60 min	TAU	STAI
Cioffi and Lubetzky (2023)	USA	Healthy	T: 15.5 (1.1) C:16.2 (1.1)	T:14/5/9 C:14/7/7	BOXVR game Length of intervention: 3 weeks Freq: 5 times a week Duration: 10 min	NT	PASF
da Silva et al. (2025)	Brazil	Healthy	T: 13.5 (0.71) C: 13.6 (0.66)	T:165/79/86 C:141/61/80	Physical activity Length of intervention: 12 weeks Freq: 2 times a week Duration: 20 min	TAU	DASS-21
Dhingra et al. (2025)	India	Mental illness (autism spectrum disorder)	NR	T:19/18/1 C:19/18/1	Aerobic exercise Length of intervention: 8 weeks Freq: 3 times a week Duration: 30 min	CON	SCARED
ElDeeb et al. (2020)	Egypt	Physical illness (premenstrual syndrome)	T: 17.3 (1.41) C: 17.9 (1.16)	T:20/0/20 C:20/0/20	Resistive exercise Length of intervention: 12 weeks Freq: 3 times a week Duration: 40 min	TAU	PMS-A
Gehricke et al. (2022)	USA	Mental illness (autism spectrum disorder)	T: 9.3 (2.0) C: 9.7 (2.2)	T:76/64/12 C:72/60/12	Physical exercise Length of intervention: 8 weeks Freq: Maximum of 3 times a week Duration: 40–50 min	CON	CBCL DSM-5 anxiety
Heidarianpour et al. (2023)	Iran	Physical illness (obesity, central precocious puberty)	T: 8.26 (0.37) C: 8.1 (0.42)	T:15/0/15 C:15/0/15	Aerobic and resistance training Length of intervention: 12 weeks Freq: 3 times a week Duration: 60 min	NT	SCAS-C
Jensen and Kenny (2004)	Australia	Mental illness (ADHD)	T: 10.63 (1.78) C: 9.35 (1.70)	T: 11/11/0 C: 8/8/0	Yoga Length of intervention: 20 weeks Freq: one time a week Duration: 60 min	CON	CPRS–R-Anxious/Shy
Kenis-Coskun et al. (2022)	Turkey	Physical illness (cystic fibrosis)	T: 9.8 (2.14) C: 10 (1.64)	T: 14/3/11 C: 14/5/9	High-intensity interval training and postural strengthening Length of intervention: 12 weeks Freq: 3 times a week Duration: NR	NT	RCADS
Khalsa et al. (2012)	USA	Healthy	T: 16.8 (0.6) C: 16.9 (0.8)	T: 74/40/34 C: 47/30/17	Yoga Length of intervention: 11 weeks Freq: 2-3 times a week Duration: 30/40 min	TAU	POMS-SF-tension/anxiety
Kuttner et al. (2006)	Canada	Physical illness (irritable bowel syndrome)	T: 14.36 (2.10) C: 13.83 (1.89)	T: 14/2/12 C: 14/6/8	Yoga Length of intervention: 4 weeks Freq: daily Duration: 10 min	WL	RCMAS
Mona et al. (2024)	India	Healthy	T: 13.44 (0.61) C: 13.54 (0.64)	T: 48/25/23 C: 46/22/24	Yoga Length of intervention: 12 weeks Freq: 3 times a week Duration: 45 min	CON	GAD-7
Nazari et al. (2020)	Iran	Physical illness (type 1 diabetes)	T: 11.22 (1.90) C: 11.00 (2.67)	T:20/NR C:20/NR	Resistance-aerobic training Length of intervention: 16 weeks Freq: 3 times a week Duration: 60 min	NT	RCMAS
Noggle et al. (2012)	USA	Healthy	T: 17.1 (0.6) C: 17.3 (0.8)	T:36/14/22 C:15/8/7	Yoga Length of intervention: 10 weeks Freq: 2-3 times a week Duration: 30/40 min	TAU	POMS-SF-tension/anxiety
Norris et al. (1992)	U.K.	Healthy	T: 16.7(NR) C: 16.7(NR)	T:14/8/6 C:16/8/8	Aerobic training Length of intervention: 10 weeks Freq: 2 times a week Duration: 25–30 min	NT	MAACL
Peens et al. (2008)	South Africa	Physical illness (developmental coordination disorder)	NR	T:20/14/6 C:17/12/5	Motor-based intervention Length of intervention: 8 weeks Freq: 2 times a week Duration: 30 min	NT	CAS
Philippot et al. (2022)	Belgium	Mental illness (depression)	T: 15.5 (1.77) C: 15.2 (1.5)	T:20/8/12 C:20/7/13	Physical exercise Length of intervention: 5–6 weeks Freq: 3-4 times a week Duration: 60 min	CON	STAI
Quach et al. (2016)	USA	Healthy	T + C: 13.18 (0.72)	T:65/24/41 C:53/25/28	Yoga Length of intervention: 4 weeks Freq: 2 times a week Duration: 45 min	WL	SCARED
Richards et al. (2014)	Uganda	Healthy	NR	T:155/74/81 C:71/71/0	Football Length of intervention: 11 weeks Freq: one time training + one time game a week Duration: 90min +40 min	WL	APAI-ALS
Rodrigues et al. (2021)	Portugal	Healthy	NR	T:34/21/13 C:36/22/14	Qigong Length of intervention: 6 weeks Freq: 1-2 times a week Duration: 15–20 min	NT	STAI
Romero-Pérez et al. (2020)	Mexico	Physical illness (obesity)	T: 10.28 (0.96) C: 9.47 (0.40)	T:54/24/30 C:51/21/30	Physical exercise Length of intervention: 20 weeks Freq: 2 times a week Duration: 50 min	NT	CMAS-R
Silva et al. (2020)	Brazil	Mental illness (ADHD)	T: 12.2 (2) C: 12 (1)	T:10/8/2 C:10/6/4	Swimming Length of intervention: 8 weeks Freq: 2 times a week Duration: 45min	NT	BAI
Syväoja et al. (2024)	Finland	Healthy	T: 8.9 (0.4) C: 8.8 (0.4)	T:127/63/64 C:132/63/69	Physical activity Length of intervention: 20 weeks Freq: 4 times a week Duration: 20 min	CON	mAMAS
Wagener et al. (2012)	USA	Physical illness (obesity)	T + C: 14 (1.66)	T:20/NR C:20/NR	Dance-based exergaming exercise Length of intervention: 10 weeks Freq: 3 times a week Duration: 60 min	WL	BASC-SRP-A-anxiety
Ziv et al. (2023)	USA	Mental illness (anorexia nervosa)	T: 15.9 (1.3) C: 16.6 (1.2)	T:7/0/7 C:8/0/8	Yoga Length of intervention: 24 weeks Freq: 2 times a week Duration: NR	TAU	STAI

T: experimental group, C: control group, NR: not reported, APAI-ALS: Acholi Psychosocial Assessment Instrument-anxiety-like syndrome, BAI: beck inventory, BASC-SRP-A-anxiety: Behavior Assessment System for Children Adolescent Self-Report Scales, CAS: child anxiety scale, CBCL DSM-5, Anxiety: Child Behavior Checklist DSM-5, anxiety subscale; CMAS-R: Manifest Anxiety Scale in Children-Revised, CON: control group with no exercise intervention, CPRS–R-Anxious/Shy: Conners’ Parent Rating Scale-Revised: Long-Anxious/Shy, DASS-21: Depression, Anxiety, and Stress Scale - Short Form, GAD-7: generalized anxiety disorder, MAACL: multiple affect adjective check list, mAMAS: the Abbreviated Math Anxiety Scale modified for children, NT: no treatment, PASF: Pediatric Anxiety Short Form 8a, PMS-A: Premenstrual syndrome questionnaire -anxiety symptoms, POMS-SF-Tension/anxiety: The Profile of Mood States short form, RCADS: Anxiety and Depression Scale in Children-Revised, RCMAS: revised child manifest anxiety scale, SCARED: screen for child anxiety related disorders, SCAS-C: Spence Children’s Anxiety Scale, STAI: State-Trait Anxiety Inventory, TAU: treatment (physical activity) as usual, WL: waitlist.

### 3.3 Risk of bias

**FIGURE 2:**
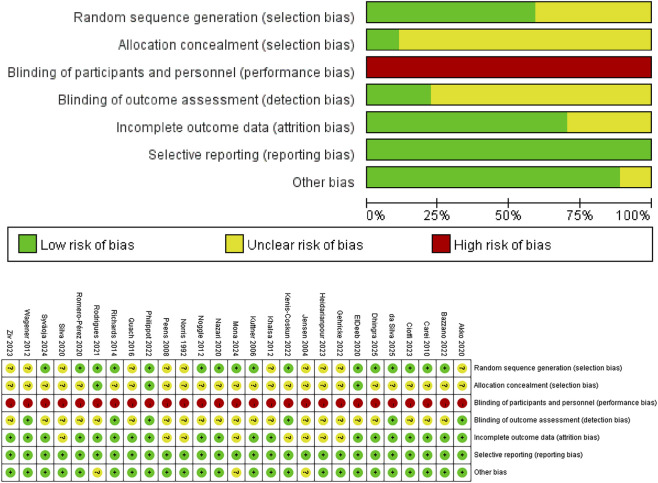
Results of cochrane risk of bias tool. Combined percentage risk of bias in each risk domain for all included trials (top).Risk of bias summaries for all exercise trials (bottom).

Concerning generation of random sequences, 16 studies that clearly described the methods were classified as low risk. The remaining 11 only mentioned randomization without specifying the method, hence were rated unclear risk. Three studies that explicitly described the allocation concealment method were deemed low risk; the rest that lacked relevant details were deemed unclear risk. Considering that exercise interventions possess specific features, blinding participants and personnel was infeasible, thus all studies were rated high risk. Six studies specifically reported implementing blinding of outcome assessment and were deemed low risk, and the rest were deemed unclear risk. 19 studies provided adequate descriptions of incomplete outcome data and were categorized as low risk, and the remaining ones were deemed unclear risk. Regarding selective reporting, all studies were classified as low risk. Three studies were deemed unclear risk regarding other bias, and the others rated low risk (Figure 2).

### 3.4 Compliance with the ACSM recommendations

In accordance with ACSM guidelines, 13 studies demonstrated an exercise adherence rate of ≥75%, meeting the criteria for high adherence, while 14 studies had an adherence rate of <75%, which were classified as low or uncertain adherence (Table 3). This discrepancy was primarily due to either a lack of detailed descriptions regarding the exercise dosage or the use of parameters that did not align with the ACSM guidelines.

**TABLE 3:** Exercise interventions evaluated according to the ACSM recommendations.

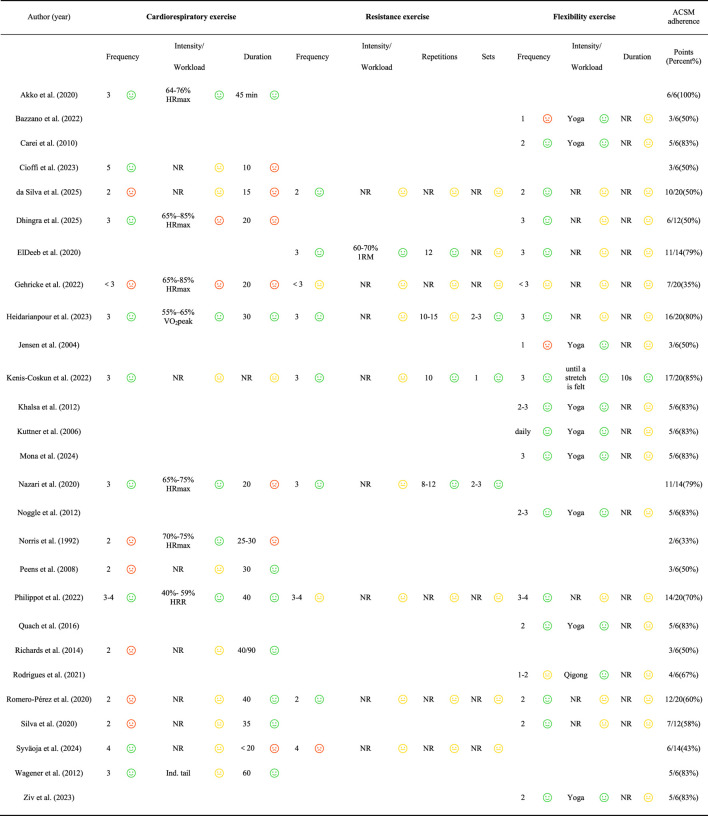

ACSM: american college of sports medicine, VO_2_peak: peak oxygen uptake, HRR: heart rate reserve, HRmax: maximal heart rate, Ind. tail: individually tailored, NR: not reported, Happy/green face: meeting the criteria (2 points), neutral/yellow face: uncertain (1 point), unhappy/red face: not meeting the criteria (0 point).

### 3.5 Meta-analysis

**FIGURE 3:**
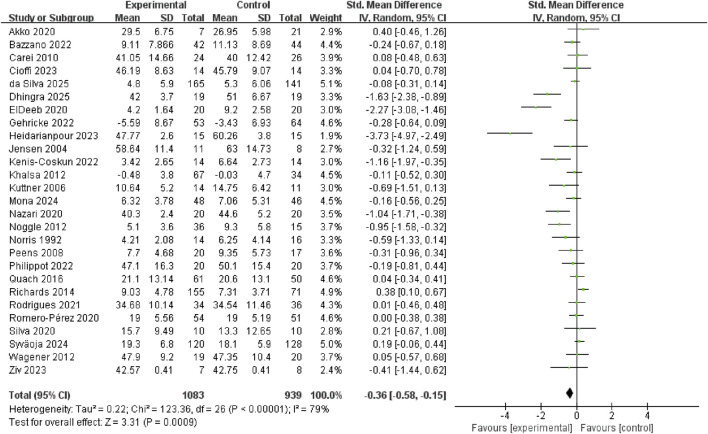
Forest plot of the effect of exercise on anxiety symptoms.

Figure 3 shows that exercise interventions may may reduce anxiety in youth (SMD = −0.36, 95% CI: −0.58 to −0.15, p = 0.0009, I^2^ = 79%). To identify potential effect modifiers, we fitted meta-regression on baseline anxiety score, mean age and proportion of boys. None of the covariates significantly predicted between-study heterogeneity: baseline anxiety (β = −0.005, 95% CI: −0.019 to 0.01, p = 0.532), mean age (β = 0.021, 95% CI: −0.077 to 0.12, p = 0.67) or proportion of boys (β = 1.059, 95% CI: −0.105 to 2.223, p = 0.075) (Supplementary Appendix S2).

**FIGURE 4:**
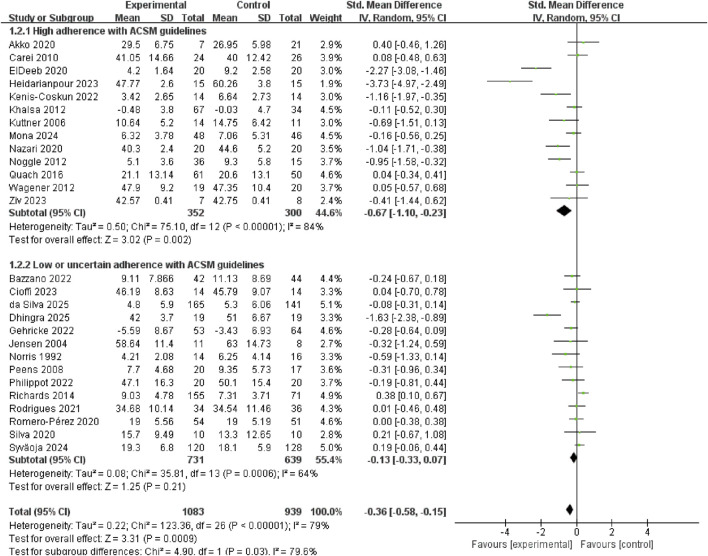
Subgroup analysis of the effect of exercise on anxiety symptoms.

Subgroup analysis revealed that anxiety reduction was significantly larger in high ACSM adherence group (SMD = −0.67, 95% CI: −1.10 to −0.23, p = 0.002. I^2^ = 84%) than in low/uncertain ACSM adherence group (SMD = −0.13, 95% CI: −0.33 to 0.07, p = 0.21, I^2^ = 64%) (Figure 4). Sensitivity analyses at 70% and 60% ACSM-adherence thresholds (two additional subgroup analyses) produced directionally consistent pooled estimates favouring exercise, while the between-subgroup interaction was significant at 70% (p = 0.03) but not at 60% (p = 0.11), indicating that the anxiolytic advantage is confined to trials achieving ≥70% compliance with ACSM guidelines and supporting the 75% threshold used in the primary analysis (Supplementary Appendixs S3, S4).

**FIGURE 5:**
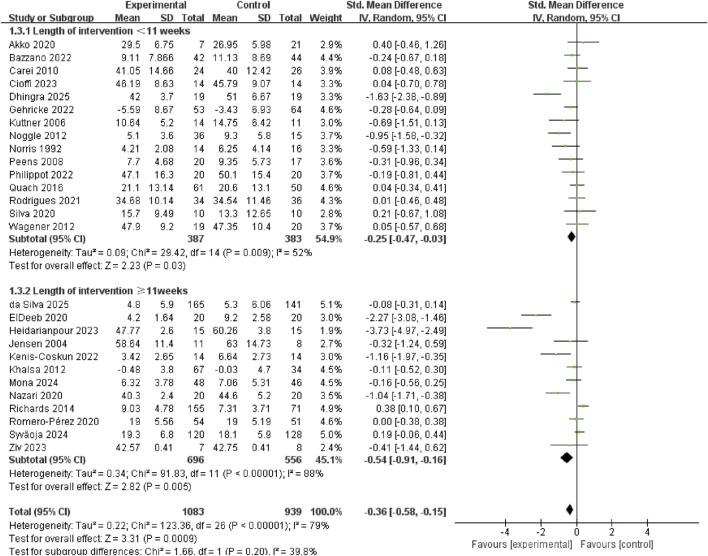
Subgroup analysis of the length of the exercise intervention.

Exercise interventions longer than 11 weeks (SMD = −0.54, 95% CI: −0.91 to −0.16, p = 0.005, I^2^ = 88%) exerted greater anxiolytic effects in youth than interventions shorter than 11 weeks (SMD = −0.25, 95% CI: −0.47 to −0.03, p = 0.03, I^2^ = 52%) (Figure 5).

**FIGURE 6:**
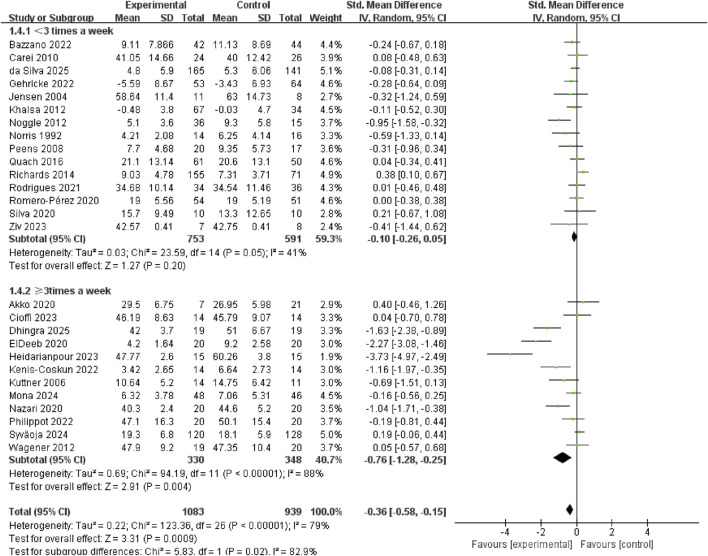
Subgroup analysis of the frequency of the exercise intervention.

Compared with interventions conducted less than 3 times/week (SMD = −0.10, 95% CI: −0.26 to 0.05, p = 0.20, I^2^ = 41%), those conducted at least 3 times/week (SMD = −0.76, 95% CI: −1.28 to −0.25, p = 0.004, I^2^ = 88%) showed significantly greater anxiolytic effects in youth (Figure 6).

**FIGURE 7:**
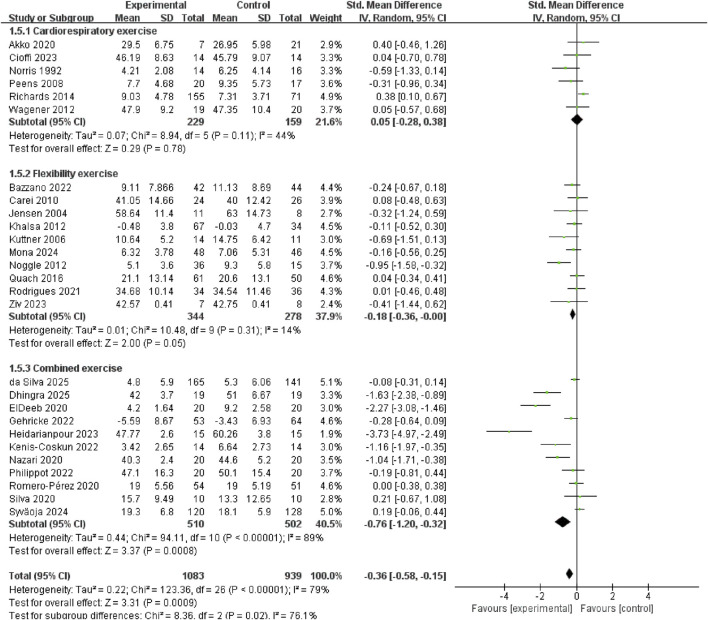
Subgroup analysis of the modalities of the exercise intervention.

Exercise modalities were classified as cardiorespiratory, flexibility or combined exercise. Compared with cardiorespiratory exercise (SMD = 0.05, 95% CI: −0.28 to 0.38, p = 0.78, I^2^ = 44%) and flexibility exercise (SMD = −0.18, 95% CI: −0.36 to −0.00, p = 0.05, I^2^ = 14%), combined exercise modalities produced larger anxiolytic effects in children and adolescents (SMD = −0.76, 95% CI: −1.20 to −0.32, p = 0.0008, I^2^ = 89%) (Figure 7).

**FIGURE 8:**
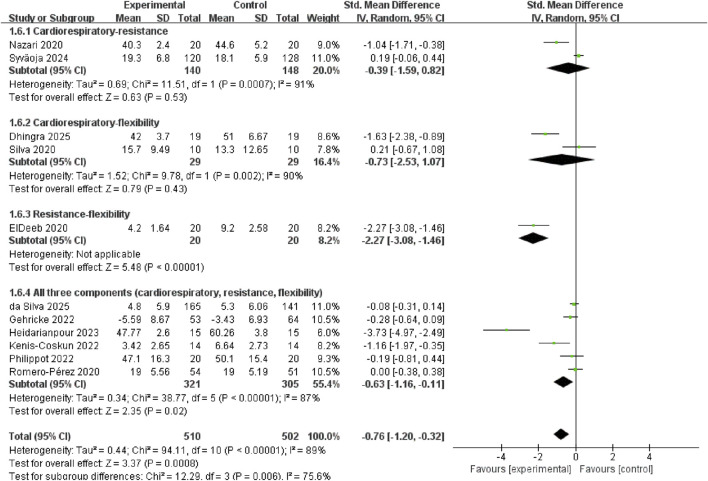
Subgroup analysis of the subtypes of the combined exercise.

To identify which specific combination of exercise components drives the anxiolytic benefit, we performed a subgroup analysis of the four subtypes of the combined exercise. The results revealed that significant reductions in anxiety were confined to resistance-flexibility (SMD = −2.27, 95% CI: −3.08 to −1.46, p < 0.00001) and all three components (SMD = −0.63, 95% CI: −1.16 to −0.11, p = 0.02, I^2^ = 87%). In contrast, cardiorespiratory-resistance (SMD= −0.39, 95% CI: −1.59 to 0.82, p = 0.53, I^2^ = 91%) and cardiorespiratory-flexibility (SMD = −0.73, 95% CI: −2.53 to 1.07, p = 0.43, I^2^ = 90%) did not reach statistical significance (Figure 8). Sensitivity analysis showed the pooled effect remained significant and directionally consistent after sequentially removing any single study (Supplementary Appendix S5).

**FIGURE 9:**
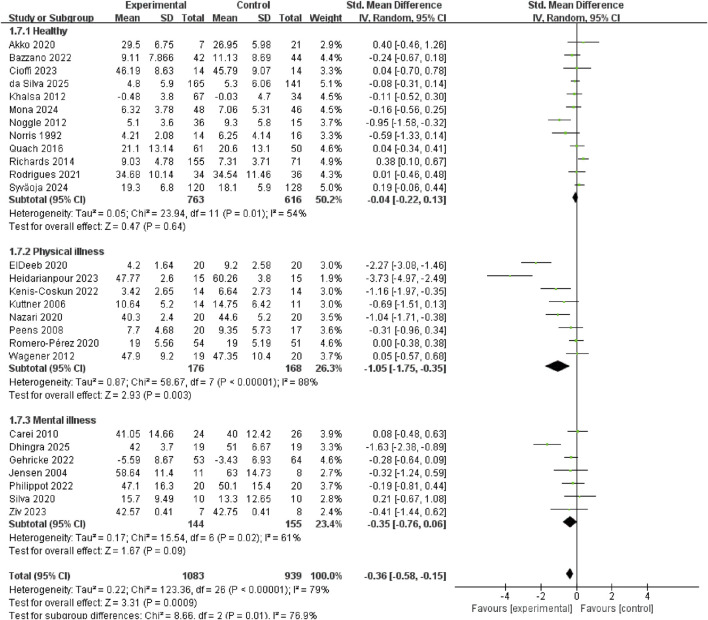
Subgroup analysis of the health status of participants.

Exercise significantly reduced anxiety symptoms in populations with physical illnesses (SMD = −1.05, 95% CI: −1.75 to −0.35, p = 0.003, I^2^ = 88%); no significant anxiolytic effects were observed in healthy populations (SMD = −0.04, 95% CI: −0.22 to 0.13, p = 0.64, I^2^ = 54%) or in individuals with mental illnesses (SMD = −0.35, 95% CI: −0.76 to 0.06, p = 0.09, I^2^ = 61%) (Figure 9).

**FIGURE 10:**
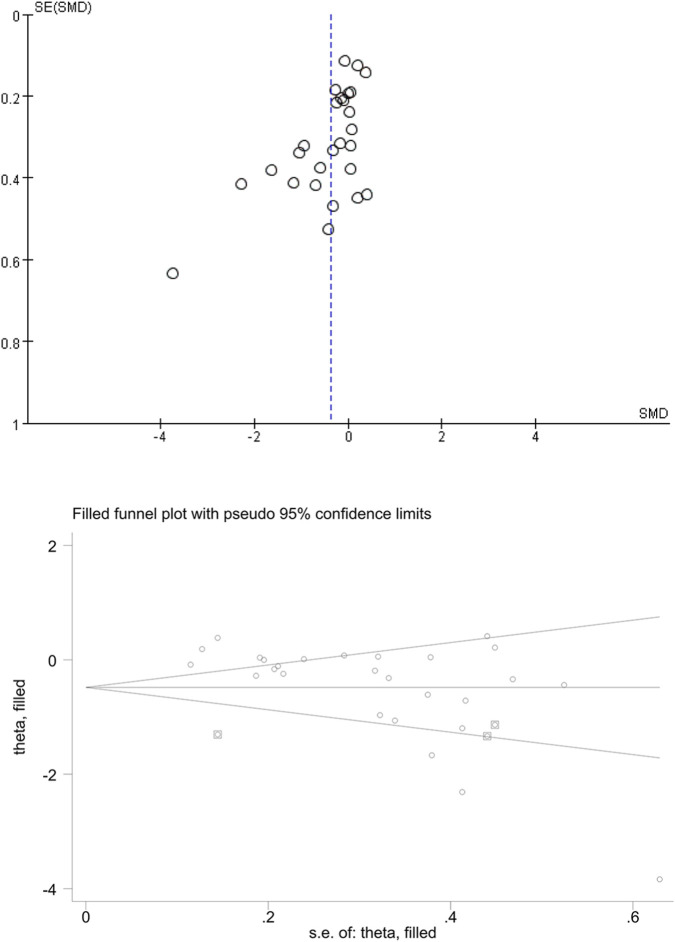
Funnel plot and trim-and-fill analysis for publication bias assessment.

Egger’s test and Begg’s test (p < 0.01) detected the publication bias, however, this bias did not affect the overall results showed by trim-and-fill analysis (Figure 10).

## 4 Discussion

This meta-analysis demonstrated that exercise interventions showed significant anxiolytic effects in children and adolescents. Moreover, the anxiety reduction was significantly larger in high ACSM adherence group compared with that in low/uncertain ACSM adherence group. Additionally, interventions lasting longer than 11 weeks were more effective than those of shorter length. Only exercise performed more than three times per week and combined exercise modalities exerted significant anxiolytic effects. Furthermore, exercise interventions were found to significantly reduce anxiety symptoms among individuals with physical illnesses.

Exercise significantly reduced anxiety symptoms in children and adolescents, consistent with meta-analyses in young adults (Carter et al., 2021; Marinelli et al., 2024). In our review, 4 of the 27 trials performed the measurement of cortisol, and only one of them found significant post-intervention reduction (Heidarianpour et al., 2023). Previous studies indicate that exercise can suppress hypothalamic-pituitary-adrenal (HPA) axis hyperactivity and regulate the release of neurotransmitters which could be translated into reduced anxiety symptoms (Fichter and Pirke, 1986; Anderson & Shivakumar, 2013; Mikkelsen et al., 2017). These mechanisms are theoretical and were not directly examined in the trials we included. Exercise may also enhance self-efficacy and improve psychological adaptation (Bodin and Martinsen, 2004). By repeatedly experiencing the sensations of fear or worry, individuals increase their familiarization with these feelings, which produces a habituation response, a mechanism similar to that underlying exposure therapy for clinical anxiety disorders (Broman-Fulks et al., 2004; Parker et al., 2018). This hypothesis remains to be further verified.

According to the ACSM guidelines, we categorized the selected studies into high and low/uncertain adherence groups (Garber et al., 2011). In line with previous studies, our two groups contained similar exercise modalities in this meta-analysis (Cui et al., 2023; Zhao et al., 2024). This analytical approach reduced the possible confounding effect caused by primary exercise modalities of ACSM compliance. A previous meta-analysis suggested that high ACSM-compliant exercise can alleviate depression in hemodialysis patients (Fang et al., 2024). Our result also indicated that exercise intervention that follows ACSM-recommended dosage can bring a significantly positive effect on reducing anxiety among children and adolescents. Therefore, in the future exercise intervention studies should follow ACSM-recommended dosage to report parameters and increase the consistency and comparability among studies.

Exercise length, frequency and modality may also influence the anxiolytic effects of physical exercise in children and adolescents. In terms of exercise length, our results demonstrated that both shorter (<11 weeks) and longer (≥11 weeks) interventions significantly alleviated anxiety symptoms in youth, and longer interventions showed better effects. In terms of exercise frequency, only the exercise interventions conducted more than 3 times per week could significantly reduce anxiety symptoms. Previous studies also indicated that different doses of exercise would exert different anxiolytic effects on college students (Ji et al., 2022; Margulis et al., 2023). Only interventions conducted longer than 8 weeks and more than 3 times/week would exert anxiolytic effects on college students (Lin and Gao, 2023). In terms of exercise modalities, the combined exercise modalities showed more anxiolytic effects. Previous study found that different exercise modalities would produce distinct effects on anxiety among adults (LeBouthillier and Asmundson, 2017). Specifically, aerobic training would reduce the psychological distress and anxiety level, while resistance exercise would heighten the sensitivity to anxiety symptoms and improve the tolerance of psychological distress (LeBouthillier and Asmundson, 2017). By integrating physical activity with mental concentration and relaxation practice, yoga and qigong would also reduce anxiety (Li et al., 2020). Moreover, significant anxiolytic effects were only observed in resistance-flexibility (one study) and all three components (cardiorespiratory, resistance, and flexibility) exercise. Neither cardiorespiratory-flexibility nor the widely advocated cardiorespiratory-resistance was found to be statistically significant, though each of the estimates was based on less than three trials and should be regarded as being preliminary. The superior efficacy of all three components (cardiorespiratory, resistance, and flexibility) exercise may reflect additive psychophysiological adaptations, enhanced parasympathetic modulation and self-efficacy. However, the limited data for any two-component combination prevents a conclusive ranking. Future studies should compare the effects of different combined exercise protocols on anxiety in children and adolescents.

Consistent with previous studies (Long and Vanstavel, 1995; Carter et al., 2021), our meta-analysis found that exercise can decrease anxiety symptoms among patients with physical illnesses. Cortisol data extracted from the eligible trials suggest that in obese girls with central precocious puberty, exercise reduced body fat and produced a sustained cortisol reduction that accompanied lower anxiety scores, indicating reversal of obesity driven HPA axis overactivity (Heidarianpour et al., 2023). In contrast, a similar psychological benefit was achieved in youths with type 1 diabetes without any additional decrease in cortisol (Nazari et al., 2020), which suggests regulation of glycaemic and autonomic systems, but not glucocorticoid inhibition. Future RCTs should combine anxiety measurements with biomarkers to elucidate these conflicting directions and prescription of precision exercises in pediatric endocrine and obesity clinics. Exercise did not significantly reduce anxiety in healthy youth or in those with mental illnesses. The failure to detect an anxiolytic effect should be interpreted cautiously. First, the scales used in most trials were developed to screen rather than to detect subtle changes, so they may lack the sensitivity needed for participants with low levels (Quach et al., 2016; Aumer and Vögele, 2025). Second, because of small samples and low event rates, the meta-analysis had insufficient power to detect small-to-moderate effects. Third, baseline heterogeneity may have obscured true effects. Healthy youth frequently presented a floor effect (Ensari et al., 2015), but those with mental illness exhibited more dispersion as a baseline which may have resulted into the underestimation of the actual effect. Thus, the absence of a significant effect in these subgroups reflects methodological constraints rather than definitive inefficacy.

There are several limitations. First, the included studies exhibited considerable heterogeneity, and the exercise dosage was not properly reported. The lack of clear definitions may have compromised the accuracy of adherence classification against ACSM guidelines. Second, because exercise interventions have unique characteristics, participant blinding was impossible, potentially biasing the results. Third, most studies assessed anxiety symptoms with self-report questionnaires, which are susceptible to both adolescent cognitive development and social-desirability bias. Fourth, objective anxiety-related biomarkers were rarely collected, which limited insight into underlying mechanisms. Fifth, insufficient detail on group separation and limited monitoring of intervention fidelity in school-based cluster trials raise the possibility of between-group contamination. Finally, the absence of uniform diagnostic criteria for anxiety, whether based on symptom cut-offs, screening questionnaires or clinician diagnosis, which limits the generalizability and cross-study comparability of findings.

Despite these limitations, this study nonetheless provides valuable implications for clinical practice. This meta-analysis not only reveals that exercise dosage is an important component of treating anxiety in children and adolescents but also facilitates the development of standardized exercise prescriptions in pediatrics, child psychiatry, and child rehabilitation. In addition, the results support using combined physical and psychological interventions for managing chronic illness in youth and using exercise-based therapy as a supportive treatment for common mental disorders.

## 5 Conclusion

This meta-analysis demonstrates that exercise interventions produce significant anxiolytic effects in children and adolescents, with larger benefits when programmes closely adhere to ACSM guidelines. Specifically, interventions lasting more than 11 weeks, delivered at least three times per week, and combined exercise modalities yield greater anxiety reduction, underscoring prescriptions readily applicable in school physical education or paediatric mental healthcare. Nevertheless, marked methodological heterogeneity and incomplete reporting of frequency, intensity, time and type hinder precise dose determination and restrict the certainty of any dose–response conclusion. Future research should adopt a standardized framework and explicit reporting standards to establish the optimal anxiolytic exercise dose.

## Data Availability

The original contributions presented in the study are included in the article/Supplementary Material, further inquiries can be directed to the corresponding author.
