# Supplementary material for: Effects of exercise based on ACSM recommendations on anxiety in children and adolescents: a meta-analysis of randomized controlled trials
Source: Front Physiol. 2026 Jan 14;16:1744254. doi: 10.3389/fphys.2025.1744254 (PMC12846938; doi:10.3389/fphys.2025.1744254)
Supplement: Supplementary file 1 [file DataSheet1.docx]

Supplementary Material

# Appendix S1. Search strategy on Pubmed.

| #1 | ((Adolescent[MeSH Terms]) OR (Child[MeSH Terms])) OR (Minors[MeSH Terms]) Sort by: Most Recent |
| --- | --- |
| #2 | ((((((((((((((((((((Adolescent[Title/Abstract]) OR (Adolescents[Title/Abstract])) OR (Adolescence[Title/Abstract])) OR (Adolescents, Female[Title/Abstract])) OR (Adolescent, Female[Title/Abstract])) OR (Female Adolescent[Title/Abstract])) OR (Female Adolescents[Title/Abstract])) OR (Adolescents, Male[Title/Abstract])) OR (Adolescent, Male[Title/Abstract])) OR (Male Adolescent[Title/Abstract])) OR (Male Adolescents[Title/Abstract])) OR (Youth[Title/Abstract])) OR (Youths[Title/Abstract])) OR (Teens[Title/Abstract])) OR (Teen[Title/Abstract])) OR (Teenagers[Title/Abstract])) OR (Teenager[Title/Abstract])) OR (Child[Title/Abstract])) OR (Children[Title/Abstract])) OR (Minors[Title/Abstract])) OR (Minor[Title/Abstract]) Sort by: Most Recent |
| #3 | (#1) OR (#2) Sort by: Most Recent |
| #4 | ((((Exercise[MeSH Terms]) OR (Sports[MeSH Terms])) OR (Circuit-Based Exercise[MeSH Terms])) OR (Endurance Training[MeSH Terms])) OR (Resistance Training[MeSH Terms]) Sort by: Most Recent |
| #5 | ((((((((((((((((((((((((((((((((((((((((((((((((((((((((((((((((((((((((((((((((Exercise[Title/Abstract]) OR (Exercises[Title/Abstract])) OR (Exercise, Physical[Title/Abstract])) OR (Exercises, Physical[Title/Abstract])) OR (Physical Exercise[Title/Abstract])) OR (Physical Exercises[Title/Abstract])) OR (Exercise, Aerobic[Title/Abstract])) OR (Aerobic Exercise[Title/Abstract])) OR (Aerobic Exercises[Title/Abstract])) OR (Exercises, Aerobic[Title/Abstract])) OR (Exercise, Isometric[Title/Abstract])) OR (Exercises, Isometric[Title/Abstract])) OR (Isometric Exercises[Title/Abstract])) OR (Isometric Exercise[Title/Abstract])) OR (Acute Exercise[Title/Abstract])) OR (Acute Exercises[Title/Abstract])) OR (Exercise, Acute[Title/Abstract])) OR (Exercises, Acute[Title/Abstract])) OR (Exercise Training[Title/Abstract])) OR (Exercise Trainings[Title/Abstract])) OR (Training, Exercise[Title/Abstract])) OR (Trainings, Exercise[Title/Abstract])) OR (Physical Activity[Title/Abstract])) OR (Activities, Physical[Title/Abstract])) OR (Activity, Physical[Title/Abstract])) OR (Physical Activities[Title/Abstract])) OR (Sports[Title/Abstract])) OR (Sport[Title/Abstract])) OR (Athletics[Title/Abstract])) OR (Athletic[Title/Abstract])) OR (Circuit-Based Exercise[Title/Abstract])) OR (Circuit Based Exercise[Title/Abstract])) OR (Circuit-Based Exercises[Title/Abstract])) OR (Exercise, Circuit-Based[Title/Abstract])) OR (Exercises, Circuit-Based[Title/Abstract])) OR (Circuit Training[Title/Abstract])) OR (Training, Circuit[Title/Abstract])) OR (Endurance Training[Title/Abstract])) OR (Training, Endurance[Title/Abstract])) OR (Resistance Training[Title/Abstract])) OR (Training, Resistance[Title/Abstract])) OR (Strength Training[Title/Abstract])) OR (Training, Strength[Title/Abstract])) OR (Weight-Lifting Strengthening Program[Title/Abstract])) OR (Strengthening Programs, Weight-Lifting[Title/Abstract])) OR (Strengthening Program, Weight-Lifting[Title/Abstract])) OR (Weight Lifting Strengthening Program[Title/Abstract])) OR (Weight-Lifting Strengthening Programs[Title/Abstract])) OR (Weight-Lifting Exercise Program[Title/Abstract])) OR (Exercise Programs, Weight-Lifting[Title/Abstract])) OR (Exercise Program, Weight-Lifting[Title/Abstract])) OR (Weight Lifting Exercise Program[Title/Abstract])) OR (Weight-Lifting Exercise Programs[Title/Abstract])) OR (Weight-Bearing Strengthening Program[Title/Abstract])) OR (Strengthening Programs, Weight-Bearing[Title/Abstract])) OR (Strengthening Program, Weight-Bearing[Title/Abstract])) OR (Weight Bearing Strengthening Program[Title/Abstract])) OR (Weight-Bearing Strengthening Programs[Title/Abstract])) OR (Weight-Bearing Exercise Program[Title/Abstract])) OR (Exercise Programs, Weight-Bearing[Title/Abstract])) OR (Exercise Program, Weight-Bearing[Title/Abstract])) OR (Weight Bearing Exercise Program[Title/Abstract])) OR (Weight-Bearing Exercise Programs[Title/Abstract])) OR (Physical training[Title/Abstract])) OR (Jogging[Title/Abstract])) OR (Running[Title/Abstract])) OR (Walking[Title/Abstract])) OR (Yoga[Title/Abstract])) OR (Qigong[Title/Abstract])) OR (Qi Gong[Title/Abstract])) OR (Dance[Title/Abstract])) OR (Tai Ji[Title/Abstract])) OR (Tai chi[Title/Abstract])) OR (Pilates[Title/Abstract])) OR (Hike[Title/Abstract])) OR (Hiking[Title/Abstract])) OR (Fitness[Title/Abstract])) OR (Swimming[Title/Abstract])) OR (Mindfulness[Title/Abstract])) OR (Cycling[Title/Abstract])) OR (Bicycling[Title/Abstract]) Sort by: Most Recent |
| #6 | (#4) OR (#5) Sort by: Most Recent |
| #7 | (Anxiety[MeSH Terms]) OR (Anxiety Disorders[MeSH Terms]) Sort by: Most Recent |
| #8 | ((((((((((((((((((((Anxiety[Title/Abstract]) OR (Angst[Title/Abstract])) OR (Nervousness[Title/Abstract])) OR (Hypervigilance[Title/Abstract])) OR (Social Anxiety[Title/Abstract])) OR (Anxieties, Social[Title/Abstract])) OR (Anxiety, Social[Title/Abstract])) OR (Social Anxieties[Title/Abstract])) OR (Anxiousness[Title/Abstract])) OR (Anxiety Disorders[Title/Abstract])) OR (Anxiety Disorder[Title/Abstract])) OR (Disorder, Anxiety[Title/Abstract])) OR (Disorders, Anxiety[Title/Abstract])) OR (Neuroses, Anxiety[Title/Abstract])) OR (Anxiety Neuroses[Title/Abstract])) OR (Anxiety States, Neurotic[Title/Abstract])) OR (Anxiety State, Neurotic[Title/Abstract])) OR (Neurotic Anxiety State[Title/Abstract])) OR (Neurotic Anxiety States[Title/Abstract])) OR (State, Neurotic Anxiety[Title/Abstract])) OR (States, Neurotic Anxiety[Title/Abstract]) Sort by: Most Recent |
| #9 | (#7) OR (#8) Sort by: Most Recent |
| #10 | ((((((Randomized) OR (Randomized Controlled Trial)) OR (RCT)) OR (Randomised)) OR (Randomised Controlled Trial)) OR (Random)) OR (Clinical trial) Sort by: Most Recent |
| #11 | (((#3) AND (#6)) AND (#9)) AND (#10) Sort by: Most Recent |

Appendix S2. Meta-Regression Results.

|  | Coef. | Std. Err. | z | P>\|z\| | [95% Conf. Interval] | |
| --- | --- | --- | --- | --- | --- | --- |
| baseline anxiety | -.0045282 | .0072537 | -0.62 | 0.532 | -.0187452 | .0096888 |
| mean age | .0214567 | .0503185 | 0.43 | 0.670 | -.0771657 | .1200791 |
| proportion of boys | 1.058765 | .5939777 | 1.78 | 0.075 | -.10541 | 2.22294 |


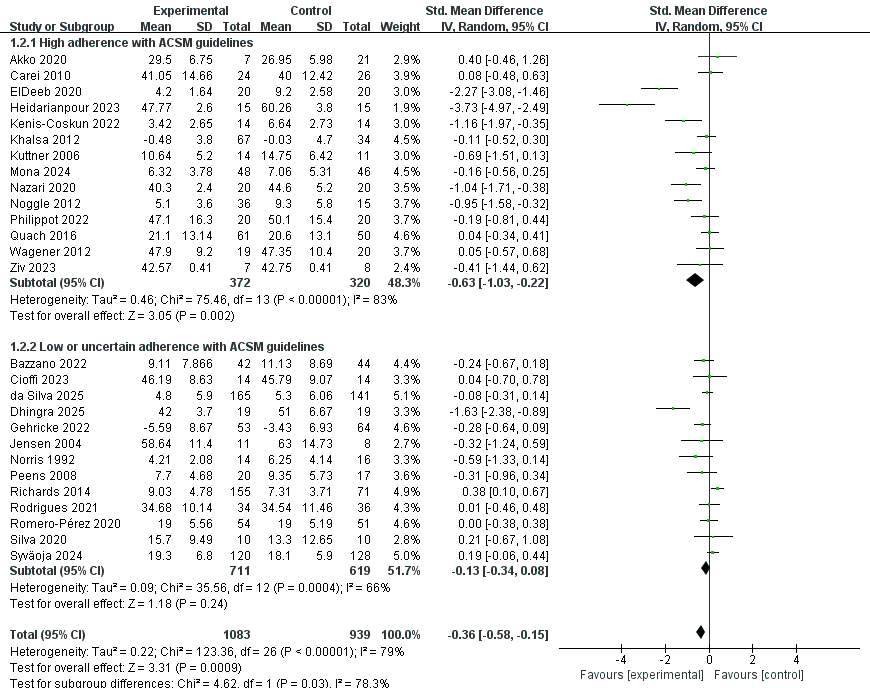


Appendix S3. Subgroup analysis of the effect of exercise on anxiety symptoms (70% ACSM-adherence threshold).


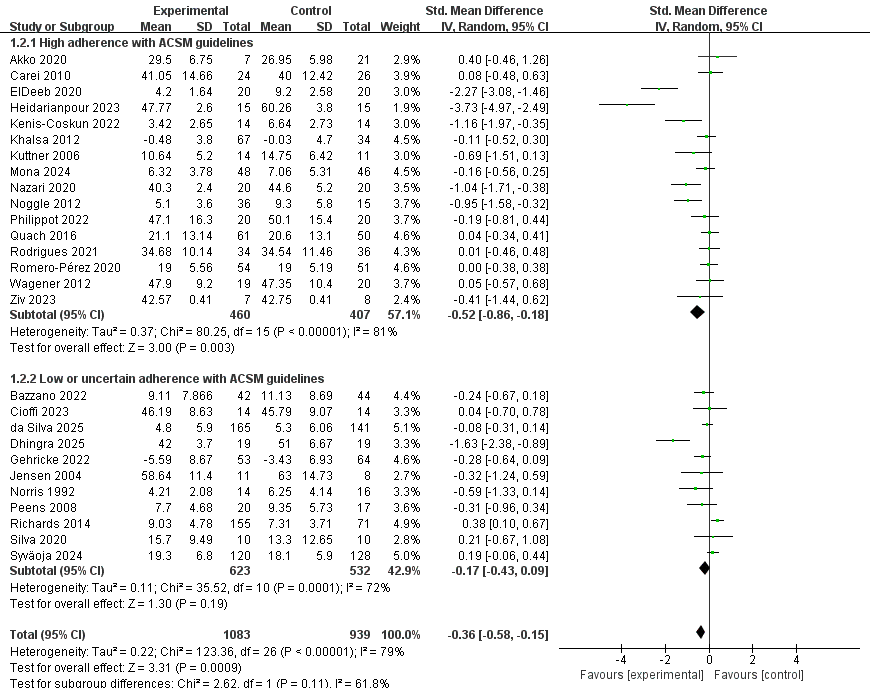


Appendix S4. Subgroup analysis of the effect of exercise on anxiety symptoms (60% ACSM-adherence threshold).

Appendix S5. Influence analysis (leave-one-out) of the combined exercise trials.
